# Polydatin Attenuates OGD/R-Induced Neuronal Injury and Spinal Cord Ischemia/Reperfusion Injury by Protecting Mitochondrial Function via Nrf2/ARE Signaling Pathway

**DOI:** 10.1155/2021/6687212

**Published:** 2021-04-20

**Authors:** Jiheng Zhan, Xing Li, Dan Luo, Wanying Yan, Yonghui Hou, Yu Hou, Shudong Chen, Jiyao Luan, Qing Zhang, Dingkun Lin

**Affiliations:** ^1^Department of Spine Surgery, The Second Affiliated Hospital of Guangzhou University of Chinese Medicine, Guangzhou 510120, China; ^2^Lingnan Medical Research Center, Guangzhou University of Chinese Medicine, Guangzhou 510405, China; ^3^Research Team on the Prevention and Treatment of Spinal Degenerative Disease, Guangdong Provincial Academy of Chinese Medical Sciences, Guangzhou 510006, China; ^4^Department of Spine, Wangjing Hospital of Chinese Academy of Chinese Medical Sciences, Beijing 100102, China; ^5^National Quality Testing Center for Processed Food, Guangzhou Inspection and Testing Certification Group Company Limited, Guangzhou 511447, China; ^6^Second Clinical College, Guangzhou University of Chinese Medicine, Guangzhou 510405, China

## Abstract

Spinal cord ischemia/reperfusion injury (SCII) is a devastating complication of spinal or thoracic surgical procedures and can lead to paraplegia or quadriplegia. Neuronal cell damage involving mitochondrial dysfunction plays an important role in the pathogenesis of SCII. Despite the availability of various treatment options, there are currently no mitochondria-targeting drugs that have proven effective against SCII. Polydatin (PD), a glucoside of resveratrol, is known to preserve mitochondrial function in central nervous system (CNS) diseases. The aim of the present study was to explore the neuro- and mito-protective functions of PD and its underlying mechanisms. An *in vitro* model of SCII was established by exposing spinal cord motor neurons (SMNs) to oxygen–glucose-deprivation/reperfusion (OGD/R), and the cells were treated with different dosages of PD for varying durations. PD improved neuronal viability and protected against OGD/R-induced apoptosis and mitochondrial injury in a dose-dependent manner. In addition, PD restored the activity of neuronal mitochondria in terms of mitochondrial membrane potential (MMP), intracellular calcium levels, mitochondrial permeability transition pore (mPTP) opening, generation of reactive oxygen species (ROS), and adenosine triphosphate (ATP) levels. Mechanistically, PD downregulated Keap1 and upregulated Nrf2, NQO-1, and HO-1 in the OGD/R-treated SMNs. Likewise, PD treatment also reversed the neuronal and mitochondrial damage induced by SCII in a mouse model. Furthermore, the protective effects of PD were partially blocked by the Nrf2 inhibitor. Taken together, PD relieves mitochondrial dysfunction-induced neuronal cell damage by activating the Nrf2/ARE pathway and is a suitable therapeutic option for SCII.

## 1. Introduction

Spinal cord ischemia/reperfusion injury (SCII) is a devastating complication of spinal or thoracic surgical procedures that often triggers a complex series of patho-physiological events [1, 2]. Although SCII can be minimized by restoring blood and oxygen supply in a timely manner, reperfusion of the ischemic tissue may lead to mitochondrial impairment, calcium overload, and energy depletion, resulting in neuronal apoptosis [3, 4]. At the cellular level, SCII is characterized by progressive degeneration or loss of spinal motor neurons (SMNs), which directly or indirectly control muscle movement [5]. Therefore, the ischemia/reperfusion (I/R) injury in spinal cord and the ensuing pathological responses can be mitigated by preserving SMNs.

Nuclear factor-E2-related factor 2 (Nrf2), a common leucine zipper transcription factor, plays a crucial role in maintaining cellular redox homeostasis. Under physiological conditions, Nrf2 is bound to its inhibitor Kelch-like ECH-associated protein 1 (Keap1) in the cytoplasm and remains in an inactive state [6]. In the presence of a stressful stimulus, Nrf2 dissociates from Keap1 and translocates to the nucleus, where it binds to the antioxidant response element (ARE) in the promoters of its target genes and transcriptionally activates the downstream detoxifying and antioxidant enzymes [7]. In addition, the Nrf2/ARE signaling pathway maintains physiological processes in the central nervous system (CNS) [8] and also plays a crucial role after CNS I/R injury. Enhanced Nrf2 activity protects against ischemic stroke injury *in vivo* by mitigating oxidative stress, apoptosis, and inflammatory response after SCII [9–11]. Thus, the Nrf2/ARE axis is a promising therapeutic target for alleviating I/R-induced neuronal damage following SCII.

**Figure 1:**
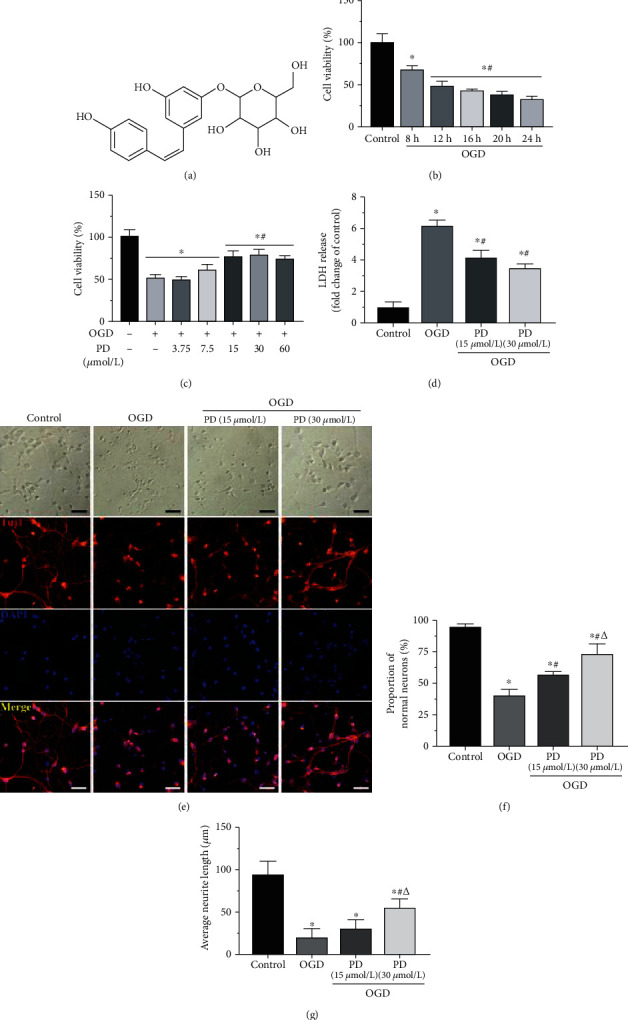
Effects of PD on the viability and morphology of SMNs exposed to OGD/R. (a) The structure of PD. (b) Percentage of viable SMNs exposed to OGD/R conditions for varying durations (8-24 h). (c) Percentage of viable SMNs with/out PD (3.75, 7.5, 15, 30, or 60 *μ*M) treatment during hypoxic injury. (d) Extracellular LDH levels in the indicated groups. (e) Representative images showing the morphology changes in the primary neurons of indicated group and the (f) number of normal neurons and (g) average neurite length. Scale bars = 50 *μ*m. ^∗^*P* < 0.05 vs. Control; #*P* < 0.05 vs. OGD; *ΔP* < 0.05 vs. OGD + PD (15 *μ*M).

Polydatin (PD, structure shown in Figure 1(a)) is a natural compound isolated from the rhizome of *Polygonum cuspidatum* and is a known activator of the Nrf2/ARE pathway [12]. In addition, PD has well-documented anti-inflammatory, antioxidant, antiapoptotic, and immunomodulatory activities [13]. Studies increasingly show that PD has neuroprotective effects as well and can protect against I/R injury of the CNS [14, 15]. Mechanistically, PD reversed the oxidative stress-induced mitochondrial dysfunction and restored the adenosine triphosphate (ATP) content in SH-SY5Y human neuroblastoma cells [16]. Consistent with this, PD also acts as a reactive oxygen species (ROS) scavenger, which is the key to inhibiting mitochondrial permeability transition pore (mPTP) opening and membrane depolarization [17]. Therefore, it is possible that PD exerts its neuroprotective effects by restoring mitochondrial function and is a promising therapeutic option for SCII.

In this study, we analyzed the protective effects of PD on SMNs using both *in vitro* and *in vivo* I/R injury models and explored the potential regulating mechanism of the Nrf2/ARE signaling pathway.

## 2. Materials and Methods

### 2.1. Usage of Animals and Ethics Statement

Healthy pregnant and male C57BL/6J mice were purchased from the Guangdong Medical Experimental Animal Center (Foshan, China). All animal experiments were performed in accordance with the NIH Guide for the Care and Use of Laboratory Animals and were approved by the Institutional Animal Care and USE Committee of the Guangzhou University of Chinese Medicine.

### 2.2. Primary SMN Cultures

SMNs were isolated and cultured as previously described [18]. Once the cells attached, the medium was replaced with neurobasal medium (Gibco, Carlsbad, CA, USA) supplemented with 2% B27 (Gibco) and 0.5 mM L-glutamine (Sigma-Aldrich, St. Louis, MO, USA). Thereafter, half of the culture medium was replaced with fresh medium every other day. The cells were identified by immunostaining for *β*-III tubulin (Tuj1), microtubule associated protein-2 (MAP-2), choline acetyltransferase (ChAT), and glial fibrillary acidic portein (GFAP). Cells on days 5-7 were used for further experiments.

### 2.3. Establishment of OGD/R Model and Treatment

The oxygen-glucose-deprivation/reperfusion (OGD/R) model was established as described by Sun et al. [19]. After washing twice with Hank's balanced salt solution (HBSS), SMNs were cultured in glucose-free Dulbecco's modified eagle medium (DMEM; Gibco) and then incubated in a hypoxic chamber with 94% N_2_, 5% CO_2_, and 1% O_2_ at 37°C for varying durations. During the reoxygenation period, the glucose-free media was replaced with complete DMEM, and the cells were cultured under normoxic conditions (75% N_2_, 5% CO_2_, and 20% O_2_) for 24 h. Control cells were not exposed to OGD/R. PD (Sigma-Aldrich, dissolved in DMSO) and brusatol (100 nM; Chengdu Herbpurify, Chengdu, China) were added to the medium at the beginning of OGD and maintained throughout the hypoxic period.

### 2.4. Assessment of Cell Viability and Cytotoxicity

Cell viability was evaluated using a cell counting kit-8 (CCK-8) assay (Beyotime, Shanghai, China) according to the manufacturer's instructions, and the absorbance at 450 nm was measured with a microplate reader (Thermo Fisher Scientific, Waltham, MA, USA). The suitably treated cells were harvested, and lactate dehydrogenase (LDH) activity was analyzed using a specific kit as instructed by the manufacturer. The optical density of each well was then determined spectrophotometrically at a wavelength of 450 nm using an automatic microplate reader.

### 2.5. Apoptosis Assays

Neuronal cell death was quantitatively assessed by terminal deoxyribonucleotidyl transferse- (TdT-) mediated biotin-16-dUTP nick-end labeling (TUNEL) assay with a Fluorescein-FragEL DNA fragmentation detection kit and Annexin V-FITC/PI double staining as reported previously [20]. The stained cells were acquired in a flow cytometer, and the percentage of apoptotic cells was analyzed.

### 2.6. Intracellular Ca^2+^ Measurement

Free intracellular Ca^2+^ ([Ca^2+^]_i_) levels were measured by staining with the fluorescence Ca^2+^ indicator Fluo-3/AM (Beyotime). The suitably treated cells were harvested, washed with PBS, and incubated with 2 *μ*M Fluo-3/AM at 37°C in the dark for 30 min. After washing twice with PBS to remove any unbound dye, the fluorescence intensity of Fluo-3/AM was measured using a microplate reader at 488 nm excitation and 525 nm emission wavelengths.

### 2.7. Detection of mPTP Opening

The opening of mPTP in the SMNs was evaluated by Calcein/AM staining in the presence of CoCl_2_ using an mPTP assay kit (Life Technologies, Carlsbad, CA, USA). Briefly, the cells were incubated with Calcein/AM and CoCl_2_ at 37°C in the dark for 40 min. The calcein that leaks out from the opened mPTP is quenched by Co^2+^, and the reduction in absorbance is measured. The stained neurons were rinsed thrice with HBSS, and fluorescence intensity was measured using a microplate reader with excitation and emission wavelengths of 488/525 nm.

### 2.8. Mitochondrial Membrane Potential (MMP/*ΔΨ*m) Assessment

The change in MMP was measured using the lipophilic cationic probe JC-1. The suitably treated cells were incubated with an equal volume of JC-1 solution (5 *μ*g/mL; Yeasen Biotech, Shanghai, China) at 37°C for 30 min. After rinsing thrice with PBS, the stained cells were observed under a confocal laser scanning microscope (Leica Microsystems, Wetzlar, Germany). Mitochondrial depolarization was evaluated in terms of the proportion of JC-1 monomers (green fluorescence at 530 nm emission) to aggregates (red fluorescence at 590 nm).

### 2.9. Measurement of ROS Levels

MitoSOX Red was used to detect the levels of mitochondrial superoxide radicals. SMNs exposed to OGD/R with/out PD treatment were incubated with 2 *μ*M MitoSOX Red (Life Technologies, Carlsbad, CA, USA) at 37°C in the dark for 20 min and washed twice with PBS. The cells were viewed under a fluorescence microscope (Olympus, Tokyo, Japan), and the fluorescence intensity was quantified using the ImageJ software (NIH, Bethesda, MD, USA).

### 2.10. Measurement of ATP Levels

ATP release was measured using the luciferase-based ATP detection assay (Beyotime) according to the manufacturer's protocol. Briefly, the suitably treated neurons were lysed and centrifuged for 5 min at 12,000 × *g*. The supernatants were aspired, and 100 *μ*L of each sample was mixed with 100 *μ*L ATP detection solution. The luminescence was measured using an automatic microplate luminometer, and the ATP level was quantified from the standard curve plotted using known concentrations (1 nM–1 *μ*M).

### 2.11. Establishment of SCII Model and Treatment

A total of 100 male C57BL/6 mice (8-10 weeks) weighing 20-25 g were randomly divided into the sham-operated, SCII model, PD-treated, and PD + Brusatol groups (*N* = 25 each). SCII was induced as previously reported with minor modifications [21]. Briefly, the aortic arch was exposed using a cervico-thoracic approach and cross-clamped between the thoracic aortic distal and left subclavian artery for 5 min to induce ischemia. After confirming ≥90% reduction in distal flow, the clamps were removed to start reperfusion. Successful induction of SCII leads to swaying of legs and slow paralysis. And animals with BBB scores higher than 2 at 24 h after I/R injury were excluded. The mice in the sham-operated group underwent the same procedure without occlusion. PD (30 mg/kg per day) was administered intragastrically for 2 days before surgery and continued until the animals were sacrificed. The Nrf2 inhibitor brusatol (2 mg/kg) was intraperitoneally (i.p.) injected once daily into mice in the PD + Brusatol group 2 h before the PD gavage. All mice were sacrificed one week postsurgery, and the spinal cords were harvested for subsequent analysis.

### 2.12. Determination of Malondialdehyde (MDA), Superoxide Dismutase (SOD), and Glutathione (GSH)

Following the manufacturer's instructions (Jiancheng, Nanjing, China) for the MDA, SOD, and GSH assays, the liquid supernatant of tissue homogenates was collected for measurement, as previously described [19]. The results were normalized to total protein content in spinal cords determined by BCA assay (Beyotime).

### 2.13. Immunofluorescence Assay

Immunofluorescence was carried out by standard protocol as previously described [22]. We used primary antibodies against Tuj1 (1 : 400; CST, Danvers, MA, USA), MAP-2 (1 : 200, CST), ChAT (1 : 200; Abcam, Cambridge, MA, USA), GFAP (1 : 500; Abcam), neuronal nuclear antigen (NeuN, 1 : 300; Abcam), Caspase-3 (1 : 400; CST), and Nrf2 (1 : 200; R&D, Minneapolis, MN, USA). Alexa Fluor® 488 donkey anti-mouse IgG (1 : 200; Invitrogen, Carlsbad, CA, USA) and Cy3-labeled goat anti-rabbit IgG (1 : 300; Invitrogen) were used as second antibodies.

### 2.14. Western Blotting

Western blotting was performed following standard methods [23]. Briefly, equal amounts of denatured protein from cellular/tissue homogenates were separated electrophoretically by SDS-PAGE and subsequently transferred onto PVDF membranes (Millipore, Bedford, MA, USA). The latter were blocked with 5% skim milk and incubated overnight with primary antibodies for Caspase-3, Bcl-2, Bax, Keap1, Nrf2, NQO1, HO-1, and cytochrome-c (Cyt-c) at 4°C. After washing thrice with buffer solution, the membranes were incubated with corresponding HRP-conjugated secondary antibodies at room temperature for 1 h. The protein bands on the membranes were visualized by enhanced chemiluminescence system (ECL) and densitometrically quantified with the ImageJ software. *β*-Tubulin served as an internal control for normalization.

### 2.15. Nissl Staining

Nissl staining was performed following standard methods. Briefly, the spinal cord tissues were fixed, cut into 5 *μ*m thick transverse sections, and embedded in paraffin. The sections were stained with 0.25% cresyl violet for 5 min to label the ventral motor neurons (VMNs).

### 2.16. Transmission Electron Microscopy (TEM)

Spinal cord specimens were fixed in 2.5% glutaraldehyde in 0.1 M cacodylate buffer for 2 h at 4°C, followed by staining with 2% osmium tetroxide. After blocking with 2% uranyl acetate, the tissue samples were dehydrated through acetone and ethanol gradient and immersed in epoxy resin. The blocks were polymerized at 60°C for 2 days and cut into ultrathin sections that were mounted on 300-mesh copper grids, counter-stained with uranyl acetate and lead citrate. All sections were examined with a JEM-1200EX electron microscope (JEOL, Tokyo, Japan).

### 2.17. Statistical Analysis

Quantitative data are expressed as mean ± standard deviation of at least three independent experiments and were analyzed statistically by one-way ANOVA or unpaired Student's *t*-test using the SPSS 24.0 software (SPSS Inc., Chicago, IL, USA). Graphs were generated by the GraphPad Prism 6.0 software (GraphPad Software, San Diego, CA, USA). *P* < 0.05 was defined as statistically significant.

## 3. Results

### 3.1. Verification of Spinal Cord Motor Neuronal Culture and PD Dose

**Figure 2:**
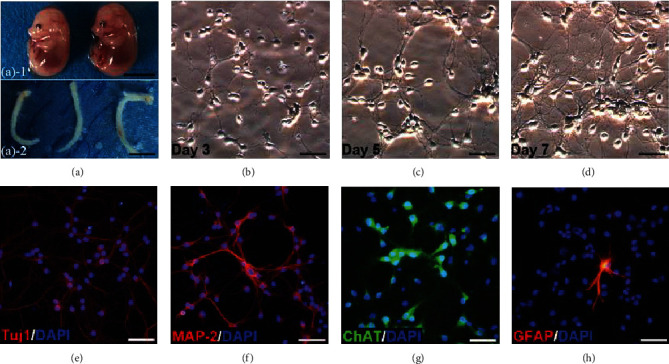
Isolation, culture, and identification of primary SMNs. (a) Spinal cords were dissected from the 14-day-old mouse embryos. Scale bars = 50 mm. Representative images of SMNs on (b) Day 3, (c) Day 5, and (d) Day 7. Primary SMNs were labeled with (e) Tuj1, (f) MAP-2, (g) ChAT, and (h) GFAP. Scale bars = 50 *μ*m.

The cells were isolated from the spinal cord of 14-day-old mouse embryos (Figure 2(a)) and cultures as per established protocols. The cells gradually enlarged, and their neurites grew longer to form vastly interconnected networks (Figures 2(b)–2(d)). Immunofluorescence showed that the majority of the cells were cholinergic neurons, while only a few were astrocytes (Figures 2(e)–2(h)). In agreement with the findings of Hong et al. [24], the viability of cultured neurons decreased in time-dependent manner after 8 to 24 h of OGD. Hypoxic exposure of 12 h decreased cell viability by 50% (Figure 1(b)) and was selected for the subsequent experiments. As shown in Figure 1(c), PD treatment significantly increased the proportion of viable cells at the doses of 15 and 30 *μ*M, which were used for all experiments.

### 3.2. PD Attenuates OGD/R-Induced Apoptosis of SMNs by Preserving Mitochondrial Function

**Figure 3:**
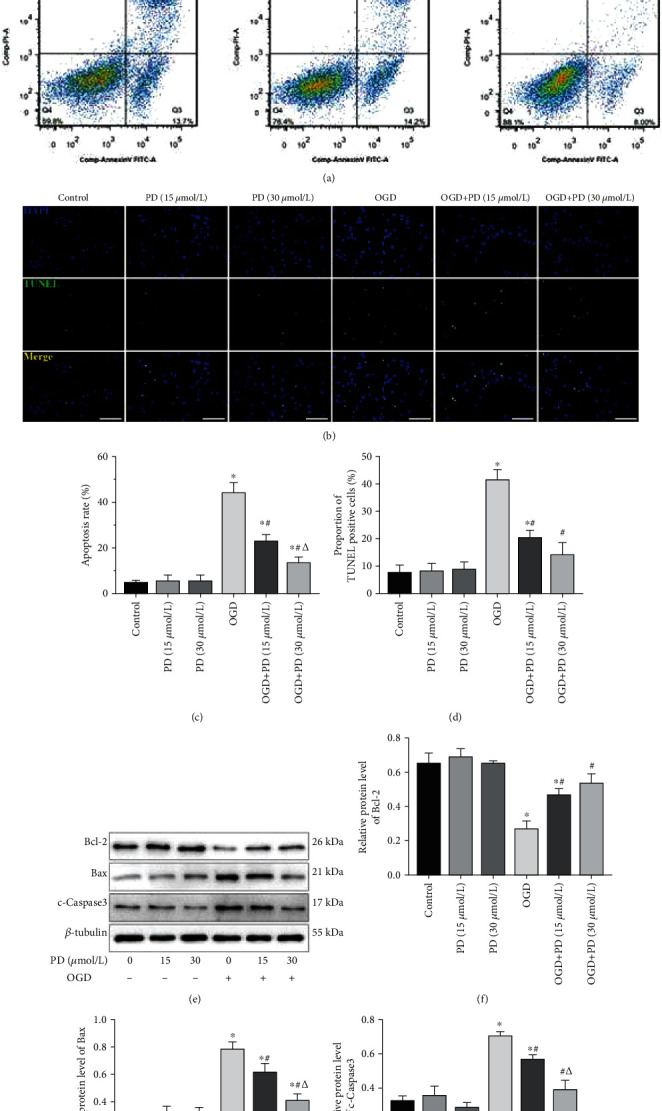
PD attenuates OGD/R-induced damage in SMNs. Cells were divided into the Control, PD (15 *μ*M), PD (30 *μ*M), OGD/R, OGD/R + PD (15 *μ*M), and OGD/R + PD (30 *μ*M) groups. (a) Representative images showing Annexin V-FITC/PI stained cells. (b) Representative images showing TUNEL-positive cells. Scale bars = 100 *μ*m. Quantification of the apoptotic cells in (c) Annexin V-FITC/PI and (d) TUNEL assays. (e) Immunoblot showing Bcl-2, Bax, and c-Caspase-3 protein levels in the neurons of the indicated groups. (f–h) Quantification of the relative protein levels. ^∗^*P* < 0.05 vs. Control; #*P* < 0.05 vs. OGD; *ΔP* < 0.05 vs. OGD + PD (15 *μ*M).

The effect of OGD/R injury and the neuroprotective effects of PD on primary cultured SMNs were evaluated in terms of secreted LDH, which is an indicator of cell membrane disintegration and cytotoxicity. OGD conditions significantly increased the LDH levels, which were restored by PD treatment in a dose-dependent manner (Figure 1(d)). Hypoxic exposure also induced morphological changes in the neurons, such as shrunken cell somas, thinner dendrites, and breakage of neuronal fibers, all of which were partially reversed by PD treatment in a dose-dependent manner (Figure 1(e)). In addition, the apoptotic ratio after OGD/R insult was as high as 43.25% ± 3.18% as indicated by Annexin V/PI staining, and significantly decreased to 20.18% ± 2.49% and 11.47% ± 2.17% in the presence of 15 *μ*M and 30 *μ*M PD, respectively. Similar results were obtained with the TUNEL assay as well (Figures 3(b) and 3(d)). Consistent with this, the antiapoptotic protein Bcl-2 was downregulated, and the proapoptotic proteins Bax and c-Caspase-3 were significantly upregulated in the neurons after OGD/R injury and markedly reversed by PD in a dose-dependent manner (Figures 3(e)–3(h)).

**Figure 4:**
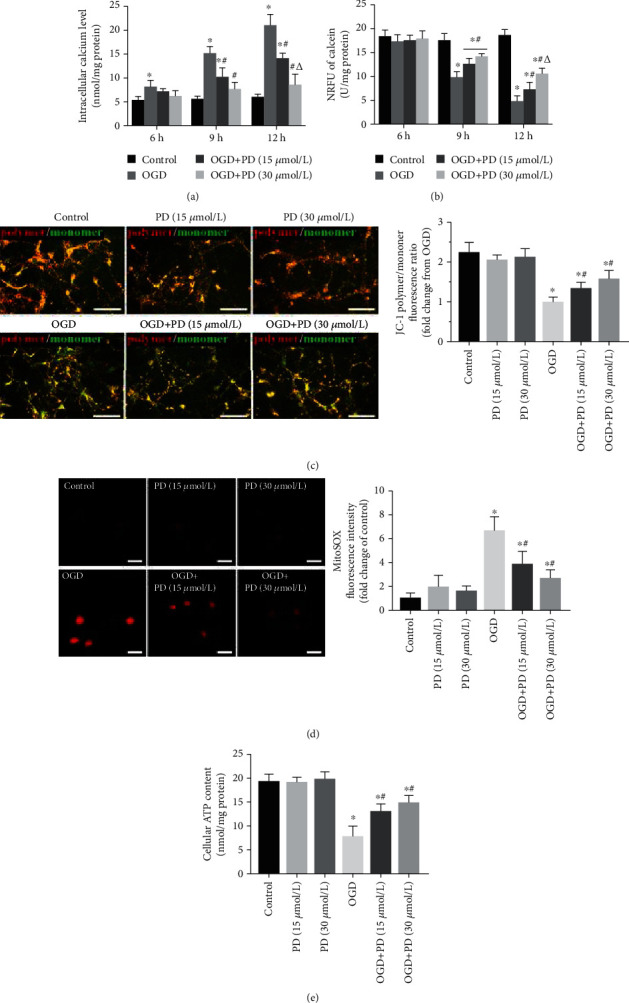
PD restores mitochondrial functions in OGD/R-injured neurons. (a) [Ca^2+^]_i_ levels in cells exposed to normoxia or hypoxia-reperfusion in the indicated groups. (b) Normalized relative fluorescence units (NRFU) of calcein indicating mPTP opening in the differentially treated cells. (c) The ratios of polymeric (red) and monomeric (green) forms of JC-1 corresponding to the MMP in the indicated groups. Scale bars = 100 *μ*m. (d) MitoSOX fluorescence intensity indicative of ROS levels. Data are presented as fold change over the Control group. Scale bars = 10 *μ*m. (e) Neuronal ATP release (nmol/mg protein) as measured by a luciferase-based assay. ^∗^*P* < 0.05 vs. Control; #*P* < 0.05 vs. OGD; *ΔP* < 0.05 vs. OGD + PD (15 *μ*M).

The mitochondria plays a critical role in hypoxia-induced neuronal apoptosis [25, 26], which is initiated by intracellular calcium dyshomeostasis and mPTP opening. The increase in Ca^2+^ levels during hypoxia triggers the opening of mPTPs, which in turn exacerbates mitochondrial dysfunction. To this end, we assessed [Ca^2+^]_i_ levels and the extent of mPTP opening. The [Ca^2+^]_i_ increased rapidly after 6 h of OGD exposure and reached its peak after 12 h of OGD exposure. However, treatment with 15 and 30 *μ*M PD decreased the [Ca^2+^]_i_ in the 12 h OGD group to 32.8% ± 3.22 and 59.52% ± 4.30, respectively (Figure 4(a)). Furthermore, hypoxic insult resulted in a gradual and significant decrease in calcein fluorescence intensity compared to the Control group, indicating increased mPTP opening in the former (Figure 4(b)). However, compared to untreated neurons (5.11 ± 1.04 U/mg protein), the normalized relative fluorescence units (NRFU) of calcein were higher in cultures treated with 15 *μ*M and 30 *μ*M PD during the 12 h OGD (8.26 ± 1.48 and 11.62 ± 1.17 U/mg protein). Thus, PD can restore intracellular calcium homeostasis and reduce the extent of mPTP opening in SMNs in response to hypoxia/reoxygenation.

To further explore the effects of PD on mitochondrial function in OGD/R-injured SMNs, we evaluated other indicators of mitochondrial activity such as MMP, ROS levels, and cellular ATP release. As revealed in Figure 4(c), OGD/R injury caused a significant decline in the *ΔΨ*m of SMNs compared to the controls, which was largely reversed by PD treatment in a dose-dependent manner. Furthermore, OGD/R also induced excessive ROS production and a sharp decrease in cellular ATP levels from 20.17 ± 1.02 to 8.64 ± 1.43 nmol/mg protein (Figure 4(d)). PD not only reduced the ROS levels but also alleviated hypoxia-induced ATP decline (Figure 4(e)). Taken together, PD protects SMNs from OGD/R injury by preserving healthy mitochondria. Since 30 *μ*m PD was most effective against OGD/R injury, this dosage was used for subsequent experiments.

### 3.3. PD Exerts Its Mito-Protective Effects in SMNs by Activating the Nrf2/ARE Signaling Pathway

**Figure 5:**
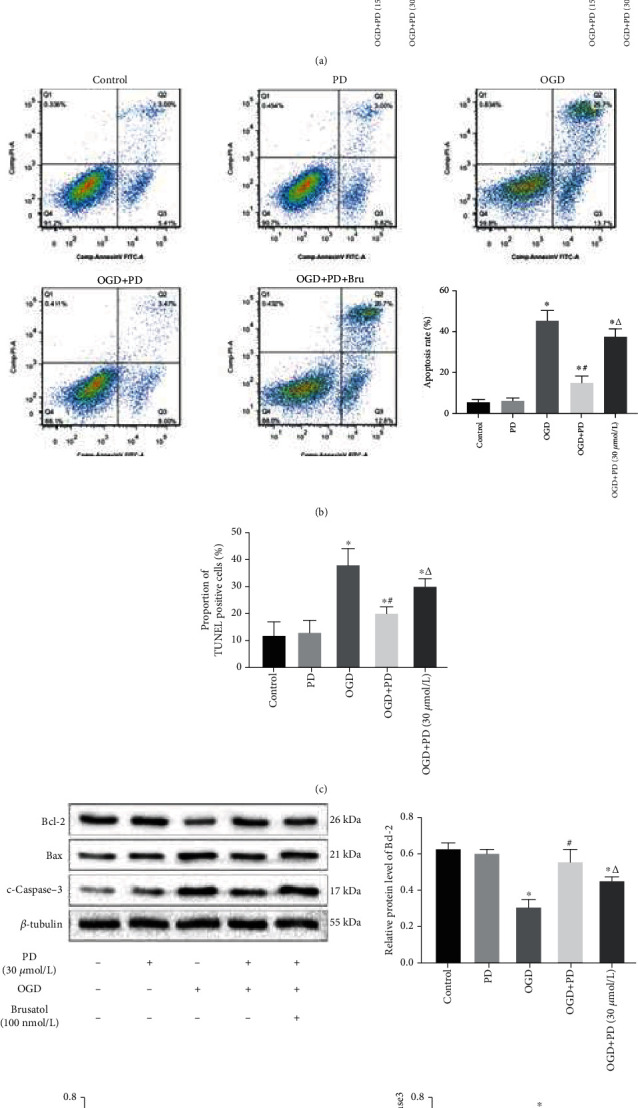
PD exerts its antiapoptotic effects by activating the Nrf2/ARE pathway. (a) Immunoblot showing levels of Keap1, Nrf2, NQO-1, and HO-1 protein in the neurons of different groups. ^∗^*P* < 0.05 vs. Control; #*P* < 0.05 vs. OGD; *ΔP* < 0.05 vs. OGD + PD (15 *μ*M). Then, cells were divided into the Control, OGD/R, OGD/R + PD, and OGD/R + PD + Brusatol groups. (b) Percentage of apoptotic cells as detected by Annexin V-FITC/PI assay. (c) Percentage of TUNEL-positive apoptotic cells. (d) Immunoblot showing Bcl-2, Bax, and c-Caspase-3 protein levels in each group. ^∗^*P* < 0.05 vs. Control; #*P* < 0.05 vs. OGD; *ΔP* < 0.05 vs. OGD + PD.

**Figure 6:**
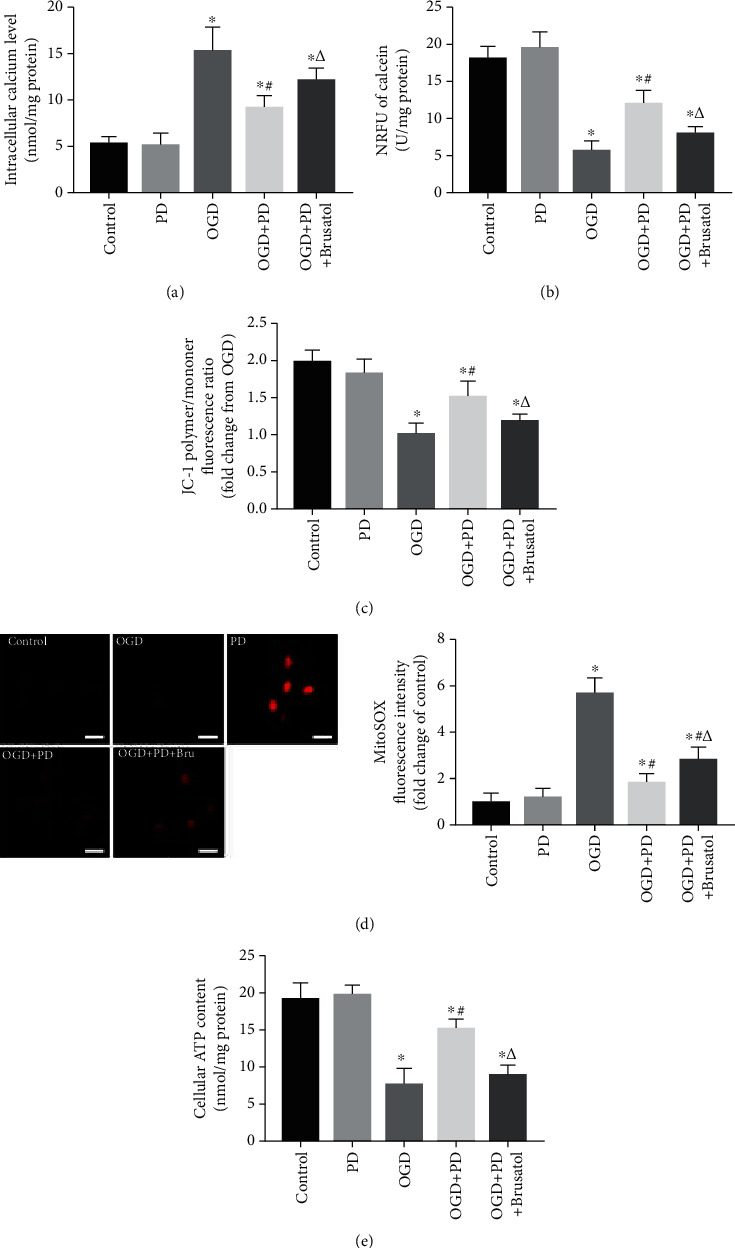
PD-mediated mito-protective depends on the Nrf2/ARE pathway. (a) [Ca^2+^]_i_ levels in cells exposed to normoxia or hypoxia-reperfusion in the indicated groups. (b) NRFU of calcein indicating mPTP opening in the differentially treated cells. (c) The ratios of polymeric (red) and monomeric (green) forms of JC-1 corresponding to the MMP in the indicated groups. (d) MitoSOX fluorescence intensity indicative of ROS levels. Data are presented as fold change over the Control group. Scale bars = 10 *μ*m. (e) Neuronal ATP release (nmol/mg protein) as measured by a luciferase-based assay. ^∗^*P* < 0.05 vs. Control; #*P* < 0.05 vs. OGD; *ΔP* < 0.05 vs. OGD + PD.

Previous studies have shown that the Nrf2/ARE signaling pathway is involved in quenching excessive ROS and reversing OGD/R-induced mitochondrial dysfunction and neuronal apoptosis [27, 28]. In addition, PD is a known Nrf2-signal activator. We found that OGD/R significantly increased Keap1 level and downregulated the Nrf2, NQO-1, and HO-1 proteins in SMNs, which was reversed by PD in a concentration-dependent manner (Figure 5(a)). Therefore, we hypothesized that the therapeutic effects of PD illustrated so far are induced by activation of the Nrf2/ARE signaling pathway. To verify this hypothesis, Nrf2 protein expression was inhibited using brusatol, which selectively regulates the level by enhances Nrf2 degradation and ubiquitination. Consistent with our hypothesis, the antiapoptotic effects of PD during OGD/R were partly suppressed by brusatol (Figures 5(b)–5(d)). Furthermore, brusatol also blocked the ameliorative effect of PD on intracellular calcium homeostasis and mPTP opening (Figures 6(a) and 6(b)). Inhibition of the Nrf2/ARE axis neutralized the ability of PD to increase MMP, scavenge ROS, and boost ATP production in the OGD-exposed SMNs (Figures 6(c)–6(e)). Taken together, PD reverses OGD/R-induced neuronal apoptosis and mitochondrial dysfunction by activating the Nrf2/ARE pathway.

### 3.4. PD Protects SMNs *In Vivo* from SCII by Reversing Mitochondrial Dysfunction

**Figure 7:**
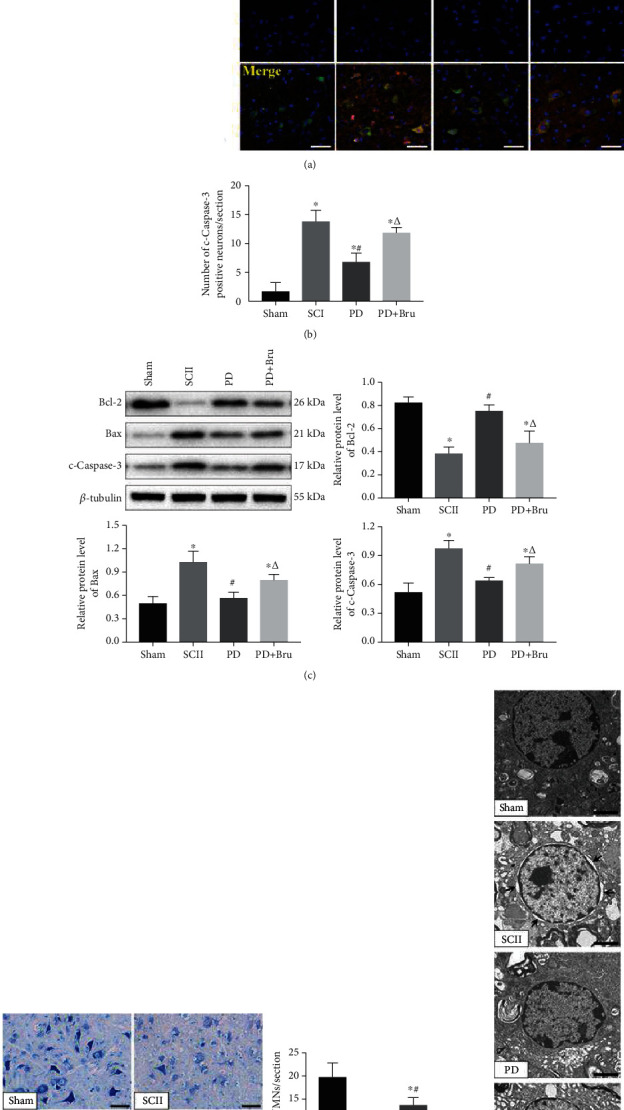
PD treatment protects SMNs from SCII-induced apoptosis. (a, b) Immunofluorescence images showing colocalization of NeuN (green) and c-Caspase-3 (red) in the spinal cord of the indicated groups. Scale bar = 50 *μ*m. (c) Quantified expression of Bcl-2, Bax, and c-Caspase-3. (d) Representative images of Nissl staining at 7 dpi and the number of VMNs. Scale bars = 50 *μ*m. (e) TEM images showing microstructures of neurons. Black arrows indicate peripheral edema and disintegrated organelles. Scale bars = 2 *μ*m. ^∗^*P* < 0.05 vs. Sham; #*P* < 0.05 vs. SCII; *ΔP* < 0.05 vs. PD.

SCII triggers extensive neuronal apoptosis and tissue damage in the spinal cords. To evaluate the potential therapeutic role of PD *in vivo*, we established a mouse model of SCII. Notably, SCII markedly increased the percentage of c-Caspase 3-positive neurons in the spinal cord tissues at 7 days post injury (dpi) compared to the sham-operated group (Figures 7(a) and 7(b)). PD treatment significantly reduced the number of apoptotic neurons in the SCII model, whereas coadministration of brusatol suppressed the beneficial effects of PD. Similar trends were observed in the levels of apoptosis-related proteins in the affected tissues of different groups (Figure 7(c)). Furthermore, PD-mediated reversal of SCII-induced VMNs loss was also partially blocked by brusatol (Figure 7(d)). Ultrastructural examination of spinal cords by TEM revealed peripheral edema and disintegrated neurons in the SCII group (Figure 7(e), black arrows). The neuronal damage recovered considerably with PD treatment, while coadministration of brusatol prevented further recovery.

**Figure 8:**
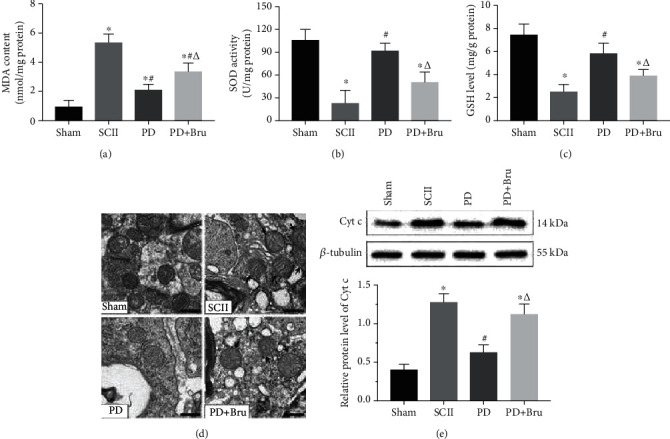
PD alleviates oxidative stress and mitochondrial dysfunction caused by SCII. (a) MDA content, (b) SOD activity, and (c) GSH levels in the spinal cords at 7 dpi. (d) TEM images showing mitochondrial ultrastructure. Arrows indicate swollen mitochondria. Scale bars = 500 nm. (e) Relative Cyt-c protein expression levels in each group. ^∗^*P* < 0.05 vs. Sham; #*P* < 0.05 vs. SCII; *ΔP* < 0.05 vs. PD.

SCII was accompanied by a significant reduction in SOD activity and GSH levels and an increase in MDA content in the affected tissues. PD administration alleviated SCII-induced oxidative stress, whereas brusatol inhibited the antioxidant capacity of PD (Figures 8(a)–8(c)). Sham-operated animals had normal mitochondria with intact membranes and cristae in the spinal neurons. However, SCII resulted in swollen and irregularly shaped mitochondria with disrupted cristae (Figure 8(d), black arrows). The mito-structural aberrations were partially alleviated by PD treatment, except in the presence of brusatol. Consistent with the above, the mitochondrial apoptotic-related protein (Cyt-c) was significantly upregulated in the spinal cords at 7 dpi and decreased after PD but not PD + brusatol treatment (Figure 8(e)). Taken together, the neuroprotective role of PD in SCII depends on its ability to mitigate oxidative stress and mitochondrial dysfunction.

## 4. Discussion

Despite recent advances in surgical techniques, patients undergoing spinal or thoracic surgery remain at high risk of postoperative complications. SCII is a life-threatening complication that can lead to permanent paraplegia due to SMN damage. Mitochondrial dysfunction is one of the key pathological events occurring early during I/R injury [29]. In addition, mitochondria play a critical role in neuronal development and maturation by modulating ATP biosynthesis, Ca^2+^ buffering, ROS production and sequestration, and apoptosis [30–32]. However, mitochondria are highly vulnerable to ischemic insult due to the concomitant deficiency of oxygen and glucose [19]. Therefore, mitochondria-targeted therapies that restore its function can potentially obviate the associated pathological conditions. Given the established neuroprotective effects of PD, we analyzed its potential role in regulating neuronal and mitochondrial injuries under I/R and explored the underlying regulatory mechanisms.

SCII leads to extensive neuronal apoptosis and necrosis in the spinal cord, which involves complex interactions among members of the Bcl-2 family of proteins (including Bcl-2 and Bax) [33, 34]. The proapoptotic protein Bax translocates from the cytosol to mitochondria and promotes apoptosis by inducing mitochondrial membrane depolarization and Cyt-c release [35]. On the other hand, the antiapoptotic Bcl-2 inhibits apoptosis by preventing the release of Cyt-c into the cytoplasm [36]. In addition, the Bcl-2 protein family maintains mitochondrial stability by modulating the Bcl-2/Bax balance [37]. Caspase-3 is a cysteine protease required for DNA fragmentation and morphological changes associated with apoptosis [38]. Consistent with previous studies, we found that OGD/R and SCII significantly downregulated Bcl-2 and upregulated Bax and c-Caspase-3. PD treatment restored the levels of the apoptosis-related proteins in a dose-dependent manner. In addition, PD also alleviated the morphological damage (peripheral edema, disintegrated organelles, and breakage of neurites) of motor neurons induced by OGD/R or SCII and decreased the proportion of apoptotic cells. A similar protective effect of PD was also observed against H_2_O_2_-induced apoptosis in BMSCs [39].

Studies increasingly show that the cytoprotective action of PD under hypoxia is related to its antioxidant activity, excitotoxicity, and inhibition of calcium ingress [39–41]. In addition, activation of the mitochondrial permeability transition (mPT) can lead to a bioenergetic, biosynthetic, and redox crisis in cells, resulting in mitochondrial dysfunction [42], which triggers neuronal apoptosis during hypoxia/reoxygenation [30]. Damaged mitochondria also produce excessive amounts of ROS that inhibit the respiratory complexes and exacerbate mitochondrial dysfunction [43]. Furthermore, ROS production triggered by OGD/R and other stresses causes morphological disintegration of neuronal mitochondria and initiates the apoptosis cascade [26]. Studies show that an increase in intracellular free Ca^2+^ levels predisposes neurons to injury, and the initiation of Ca^2+^-dependent mPT in particular is associated with hypoxia-induced apoptosis [44, 45]. PD modulates [Ca^2+^]_i_ in ventricular myocytes and mast cells [46, 47] and also attenuated [Ca^2+^]_i_ elevation in SMNs undergoing hypoxia/reoxygenation. Furthermore, PD protected SMNs from OGD/R-induced apoptosis by suppressing Ca^2+^-dependent mPT. It also alleviated loss of MMP in the cells exposed to OGD/R, as well as SCII-induced mitochondrial injury (apparently swollen with poorly defined cristae) *in vivo* and blocked the Bcl-2/Cyt-c apoptotic pathway.

**Figure 9:**
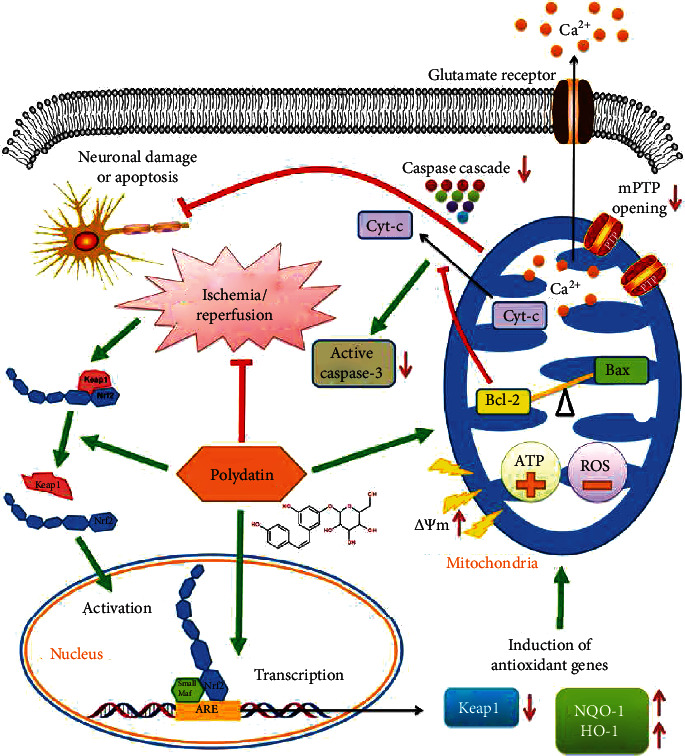
Mechanistic basis of the neuroprotective action of PD in SCII. PD promotes the nuclear translocation of Nrf2 and activates Nrf2/ARE singling, thereby inducing the ARE-driven genes NQO-1 and HO-1 that attenuate mitochondrial dysfunction. It also restores the Bcl-2/Bax balance and blocks Cyt-c-initiated Caspase cascade, which prevents neuronal damage and apoptosis.

The mPT depends on the opening of mPTPs located at the junction between the outer and inner mitochondria membranes [48]. The mPTPs of the inner mitochondrial membranes are normally closed but can open following I/R injury, allowing medium-sized molecules to move freely across the membrane [49, 50], which eventually leads to mitochondrial swelling and relocation of apoptogenic substances [51]. Therefore, mPTP opening is regarded as a critical determinant in the genesis of mitochondrial dysfunction and apoptosis. The stilbenoid compound resveratrol protects mitochondria and prevents I/R injury by targeting mPTP through translocation of GSK-3*β*, which ultimately interacts with cyclophilin D to regulate membrane permeability [52]. Likewise, its glucoside PD also significantly inhibited mPTP opening in the injured SMNs. A major consequence of hypoxia/reoxygenation injury-triggered mitochondrial dysfunction is ATP depletion [19]. PD effectively scavenged mitochondrial ROS and restored ATP production in the OGD/R neurons. Thus, PD-mediated neuroprotection of PD is mainly due to prevention of mitochondrial injury and renewal of its functions. Mitochondrial apoptosis is regulated by multiple factors and signaling pathways. The ubiquitously expressed Nrf2 is a key mediator of the antioxidant response via its interaction with ARE [53]. The Nrf2/ARE transcriptional pathway is the master regulator of multiple patho-physiological processes, including oxidative stress, inflammation, and mitochondrial dysfunction. In addition, coordinated upregulation of ARE-driven genes protects organs from I/R injury [54, 55]. A recent study showed that the antioxidant MitoQ alleviated renal I/R injury by promoting Nrf2-mediated mitophagy [56]. Furthermore, Nrf2/ARE activation has been shown to reduce mitochondrial dysfunction after traumatic brain injury in mice [57]. However, few studies have focused on the links between SMNs apoptosis, SCII, mitochondrial dysfunction, and Nrf2/ARE signaling. To the best of our knowledge, this is the first to report that activation of the Nrf2/ARE pathway attenuates I/R injury-induced SMNs apoptosis by preserving functional mitochondria (Figure 9). In addition, the protective effects of PD were neutralized by the Nrf2 inhibitor brusatol, indicating that the therapeutic action of PD is dependent on the Nrf2/ARE axis.

In conclusion, PD can attenuate hypoxia/reoxygenation-induced neuronal injury *in vitro* and *in vivo* by restoring mitochondrial function by activating the Nrf2/ARE signaling pathway and is a promising therapeutic agent against SCII.

## Data Availability

The datasets used to support the findings of this study are available from the corresponding author upon request.

## Abbreviations

CNS: Central nervous system
SCII: Spinal cord ischemia/reperfusion injury
PD: Polydatin
OGD/R: Oxygen–glucose-deprivation/reperfusion
SMNs: Spinal cord motor neurons
TUNEL: Terminal deoxyribonucleotidyl transferse- (TdT-) mediated biotin-16-dUTP nick-end labeling
mPTP: Mitochondrial permeability transition pore
MMP: Mitochondrial membrane potential
ROS: Reactive oxygen species
ATP: Adenosine triphosphate
LDH: Lactate dehydrogenase
MDA: Malondialdehyde
SOD: Superoxide dismutase
GSH: Glutathione
TEM: Transmission electron microscopy
Keap1: Kelch-like ECH-associated protein 1
Nrf2: Nuclear factor E2-related factor 2
ARE: Antioxidant response element
NQO-1: NAD(P)H: quinine oxidoreductase-1
HO-1: Heme oxygenase-1.
